# HMGB1 in Amyloid-Beta Pathology: Mechanistic Insights and Therapeutic Implications for Alzheimer’s Disease

**DOI:** 10.3390/ijms27198681

**Published:** 2026-09-28

**Authors:** Song-I Seol, Ji-Eun Kim, Dashdulam Davaanyam, So-Young Park, Eun-Hwa Lee, Pyung-Lim Han, Tae-Kyung Kim, Ja-Kyeong Lee

**Affiliations:** 1 Department of Anatomy, Inha University School of Medicine, Incheon 22212, Republic of Korea; siseol@inha.ac.kr (S.-I.S.);; 2 Department of Physical Education and Sport Science Institute, Korea National Sport University, Seoul 05541, Republic of Korea; 3 Neuromed R&D Institute, Neuromed Inc., Rm 605 Industry-Academic Cooperation Building-B, 1071, An yang cheon-ro, Yangcheon-gu, Seoul 07953, Republic of Korea; so97072003@nate.com (S.-Y.P.);; 4 Ewha Medical Research Institute, College of Medicine, Ewha Womans University, Seoul 07985, Republic of Korea; 5 Department of Brain and Cognitive Sciences, Scranton College, Ewha Womans University, Seoul 03760, Republic of Korea

**Keywords:** HMGB1, neuroinflammation, microglia, DAMP, Aβ pathology

## Abstract

Alzheimer’s disease (AD) is a progressive neurodegenerative disorder characterized by the accumulation of amyloid-beta (Aβ) plaques and neurofibrillary tangles. Beyond these hallmark pathological features, the complex pathogenesis of AD involves cellular dysfunctions associated with chronic oxidative stress and neuroinflammation. Central to these inflammatory pathways is the extracellular release of the nuclear protein high-mobility group box 1 (HMGB1) from neurons and microglia. Extracellular HMGB1 disrupts homeostatic and reparative microglial functions, impairing the phagocytic clearance of amyloid-beta and driving neuroinflammation to accelerate AD pathogenesis. Consequently, targeting HMGB1 offers a promising therapeutic strategy for AD. This review examines recent progress and key insights regarding HMGB1-associated neuroinflammatory pathology in AD. We evaluate its regulation across transcriptional upregulation, cellular redistribution, age-related alterations, and Aβ-associated pathology. We further detail the pleiotropic effects of extracellular HMGB1, including neuroinflammation, defective Aβ phagocytic clearance, and genomic instability. Finally, we discuss the potential clinical utility of targeting HMGB1 to mitigate AD progression and provide HMGB1-related perspectives for future studies.

## 1. Introduction

Alzheimer’s disease (AD) is a progressive neurodegenerative disorder characterized by the accumulation of extracellular amyloid-beta (Aβ) plaques and intracellular neurofibrillary tangles [1]. While these aggregates represent the pathological hallmarks of the disease, AD pathogenesis also involves a complex array of neural cell dysfunctions, associated with oxidative stress, neuroinflammation, and trans-vascular efflux [2].

The amyloid cascade hypothesis posits that accumulated Aβ is the primary driver of pathological changes in AD brains. Oligomeric or fibrillar Aβ activates microglia, triggering chronic neuroinflammatory responses [2,3]. Among these changes, the Aβ-induced release of the alarmin high-mobility group box 1 (HMGB1) from both neurons and microglia is particularly notable [4,5]. Once secreted, extracellular HMGB1 exacerbates pro-inflammatory responses and impairs the homeostatic reparative functions of microglia, exacerbating AD pathogenesis [6,7]. Furthermore, HMGB1 exhibits a binding affinity for Aβ and co-localizes within senile plaques [4,8]. Given these complex biological interactions, elucidating the pathological contributions of HMGB1 in the brain throughout AD pathogenesis is essential for understanding the underlying pathology and developing targeted therapeutic strategies.

While numerous reviews have summarized the molecular structure of HMGB1 and its multifaceted cellular functions across neuronal and non-neuronal contexts [9,10,11,12], its specific role in AD pathogenesis remains understudied. In this review, we outline HMGB1’s cellular functions and inflammatory roles in the context of Aβ pathology. Specifically, we highlight HMGB1’s actions on microglia, focusing on their shift between homeostatic and reparative functions and neuroinflammatory roles. In addition, we address key conceptual ambiguities in the field and discuss the therapeutic potential of targeting HMGB1 signaling in AD. Section topics encompass fundamental HMGB1 biology while extending to emerging, understudied concepts in AD. The relevant literature published over the past two decades was identified through iterative searches of PubMed and Web of Science covering major advancements in HMGB1, Aβ pathology, neuroinflammation, and microglial responses. Inclusion focused primarily on peer-reviewed original research examining HMGB1 in Aβ pathology, while critical review articles were included to address HMGB1 molecular structure and established cellular mechanisms in non-neural fields. To identify potential therapeutic agents and biologics targeting HMGB1 and HMGB1-related biomarkers, secondary searches were conducted across PubMed, patent databases, and Google search using combinations of ‘HMGB1’, ‘A-box’, ‘B-box’, ‘peptides’, ‘siRNA’, ‘in vivo’, ‘therapy’, ‘microglia’, ‘inflammation’, ‘animal models’, and/or ‘AD’.

## 2. Molecular Structure and Dynamics of Cellular Function of HMGB1

### 2.1. Molecular Structure of HMGB1

**Figure 1:**
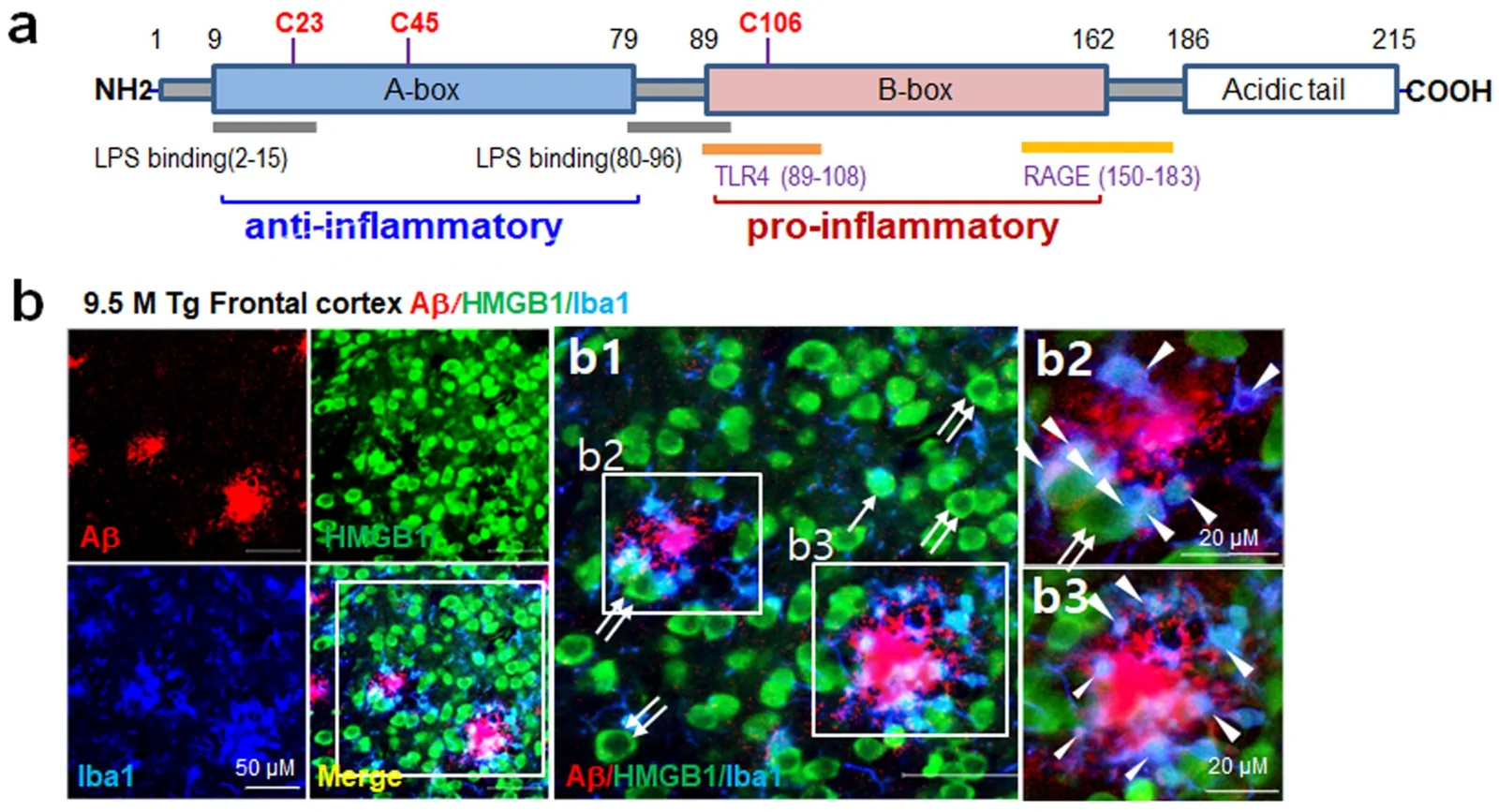
HMGB1 domain structure and histological localization in the brain of APPswe/PS1dE9 mice. (**a**) Schematic structure of HMGB1, consisting of two N-terminal DNA-binding domains (A box and B box) and an acidic C-terminal tail. Individual A-box and B-box peptides are known to exhibit anti- and pro-inflammatory activities, respectively. The three redox-sensitive cysteine residues (C23, C45, and C106) and the binding sites for LPS, TLR4, and RAGE are noted. (**b**) Triple immunofluorescence staining of Aβ plaques (red, anti-Aβ42), HMGB1 (green, anti-HMGB1), and microglia (deep blue, Iba1) in the frontal cortex of Tg-APP/PS1 mice (9.5 months). Arrows and double arrows in (**b1**) indicate HMGB1 immunoreactivity in nucleus and cytoplasm, respectively, of neurons (HMGB1-positive/Iba1-negative cells). Arrowheads in (**b2**,**b3**) indicate HMGB1 immunoreactivity in microglia (Iba1-positive) surrounding Aβ plaque. Scale bars; 20 or 50 µm. (**b1**–**b3**) are adapted from ref. [5].

HMGB1 is a highly conserved, 215-amino-acid non-histone nuclear protein in mammals. It consists of three distinct structural domains: two DNA-binding domains, the A-box (residues 9–79) and B-box (residues 89–162), and a negatively charged, acidic C-terminal tail (residues 186–215) (Figure 1a). Full-length HMGB1 acts as a DNA chaperone that binds and bends DNA to regulate transcription and nucleosome stability [9,10]. As a peptide fragment, the A-box acts as a competitive antagonist against full-length HMGB1. Accordingly, this A-box peptide is frequently deployed as a therapeutic agent to block HMGB1-mediated pathology. On the contrary, the B-box fragment is regarded as the domain responsible for its proinflammatory functions. The C-terminal acidic tail facilitates interactions with histones and regulates DNA bending [9,10].

### 2.2. HMGB1: Playing a Dual Role as a Nuclear Protein and Extracellular DAMP

HMGB1 is localized predominantly within the nucleus under normal physiological conditions, anchored by two nuclear localization signals: NLS1 (residues 28–44) and NLS2 (residues 179–185). While in the nucleus, it acts as a DNA chaperone to maintain chromosomal architecture and genomic stability, HMGB1 also serves as a cytosolic nucleic acid sensor, triggering innate immune responses [13] and enhancing autophagy [14]. Upon secretion extracellularly, HMGB1 acts as a damage-associated molecular pattern (DAMP), triggering local inflammatory and systemic immune responses. Immunohistochemical data revealed that HMGB1 is widely expressed in the nuclei of neurons, microglia, and astrocytes in healthy brains; in contrast, its location is notably shifted to the cytosol and extracellular space in the brains of AD transgenic animals (Figure 1b). Aβ directly stimulates HMGB1 secretion in primary cortical neurons, with monomers and oligomers eliciting significantly greater secretion than fibrils [4]. Aβ oligomer-induced HMGB1 release was also demonstrated not only in primary cortical neurons but in primary microglia [5], while Aβ25-35 also induced HMGB1 release in primary hippocampal cultures [15]. These reports suggest that various Aβ species induce HMGB1 release with varying potencies in primary neuronal and microglial cultures. Although the exact mechanisms behind these differences remain unclear, they may be attributed to variations in receptor dependency and cytotoxicity.

Because HMGB1 lacks a classical signal peptide, its secretion bypasses the canonical ER–Golgi network, relying instead on cell-type- and context-dependent secretory pathways. In macrophages and monocytes, HMGB1 is secreted via secretory lysosomes [16,17], whereas it utilizes alternative unconventional pathways including secretory autophagy and specialized vesicular trafficking networks in HEK293T cells [18].

Divergent pathways are involved in the nucleo-cytoplasmic translocation of HMGB1. HMGB1 undergoes post-translational modifications, such as acetylation, phosphorylation, and methylation, which alter its NLSs and weaken its affinity for nuclear DNA. Specifically, acetylation of the two lysine-rich NLSs disrupts its affinity for chromatin, initiating its nuclear export [17]. The acetylation and deacetylation of NLS1 and NLS2 are mediated by histone acetyltransferase (HAT) and histone deacetylase (HDAC) family proteins, respectively [17]. Other post-translational modifications such as phosphorylation [19] and ADP-ribosylation [20] also induce the nucleo-cytosolic translocation and extracellular release of HMGB1. Growing evidence indicates that the Sirtuin (SIRT) family plays a critical role in this regulatory process. SIRT1 deacetylates HMGB1, thereby promoting its nuclear retention and preventing its release [21]. Conversely, SIRT2 physically interacts with and deacetylates the K43 residue within the NLS1 of HMGB1, a modification that enhances their interaction and drives its cytoplasmic localization [22]. Thus, SIRT1 and SIRT2 act as opposing regulators of HMGB1 translocation. Importantly, in the aging brain, SIRT1 is typically downregulated while SIRT2 is upregulated, suggesting that an altered SIRT1:SIRT2 balance may facilitate HMGB1-mediated neuroinflammatory pathways associated with age-related cognitive decline [23].

Following inflammatory stimulation or cellular injury, HMGB1 rapidly translocates from the nucleus to the cytosol. Indeed, fluorescence immunohistochemistry demonstrates a marked enrichment of HMGB1 within the cytoplasm of neurons [4,5] and microglia [5,8] of transgenic AD mice. Within the cytosol, HMGB1 regulates autophagy by activating Beclin-1 [14], and promotes mitophagy following traumatic brain injury [24]. Kim et al. demonstrated that cytosolic HMGB1 mediates LPS-induced microglial autophagy through its interaction with NOD2 (nucleotide-binding oligomerization domain-containing protein 2), thereby suppressing proinflammatory responses [25]. However, it was also reported that extracellular HMGB1-mediated activation of RAGE signaling impaired mitophagy and triggered inflammatory responses in microglia [26]. Thus, HMGB1 exerts opposing effects on autophagy depending on its cellular context. At present, whether the cytoplasmic accumulation of HMGB1 in AD-like brains is likely a transient step toward extracellular release or fulfills a distinct intracellular role remains unclear and warrants further investigation.

### 2.3. Redox-Sensitive Regulation of HMGB1 Secretion and Its Action

In AD brains, Aβ-mediated changes trigger profound oxidative stress in both neurons and microglia. Beyond post-translational modifications, oxidative stress may act as a critical driver of HMGB1 release, particularly under pathogenic conditions of neurodegenerative diseases.

HMGB1 contains three conserved cysteine residues: C23, C45, and C106 that serve as a dynamic redox switch, governing its extracellular activities and dictating downstream inflammatory pathways under pathological conditions. Disulfide HMGB1 (dsHMGB1), containing a C23–C45 bond, binds TLR4 to induce cytokine production in macrophages [27]. Conversely, all-thiol HMGB1 (fully reduced) forms a hetero-complex with CXC-chemokine ligand 12 (CXCL12) to stimulate the C-X-C motif chemokine receptor 4 (CXCR4) receptors to mediate migration of inflammatory cells [28]. Finally, fully oxidized sulfonyl HMGB1 fails to bind either receptor [27]. Notably, these interactions are strictly redox-dependent. The formation of an intramolecular disulfide bond, catalyzed by peroxiredoxins I and II (PrxI/II), yields dsHMGB1, which is both necessary and sufficient for the nucleocytoplasmic translocation and secretion of HMGB1 [29]. Aging brains exhibit profoundly enhanced oxidative stress, in part due to cumulative changes caused by stress hormones [30,31,32]. This is further exacerbated in AD brains, which are characterized by heightened protein, lipid, and DNA oxidation, as well as glycoxidation [33,34,35]. Given that AD brains are characterized by chronic oxidative stress, dsHMGB1 likely accumulates to act as a key pathogenic mediator in AD pathogenesis. Accordingly, characterizing the physiological significance of these distinct HMGB1 redox states in the AD brain and delineating their modulation of Aβ pathogenesis will be critical for identifying and developing target-specific therapeutics.

### 2.4. Extracellular HMGB1 as a Pleiotropic Regulator of Immune Cell Homeostasis

Once secreted, extracellular HMGB1 elicits diverse cellular responses by interacting with an array of cell-surface receptors. These include pattern recognition receptors such as TLR2/4, RAGE, CXCR4, TIM-3 (HAVCR2), and Siglec-10 [27,28,36,37,38], as well as integrins and co-receptors including CD11b/Mac-1, CD14, and Fc receptors [39,40]. While these interactions may require distinct multiple structural motifs, only a few functional domains within HMGB1 have been characterized. The TLR4-binding domain (residues 89–108) resides within the B-box (residues 89–162), while the RAGE-binding domain is located between residues 150 and 183, spanning the C-terminal region of the B-box and extending beyond it (Figure 1a).

In contrast to its established proinflammatory roles, growing evidence suggests that extracellular HMGB1 can exert anti-inflammatory effects, promoting tissue regeneration and wound healing through distinct receptor networks. Specifically, extracellular HMGB1 exhibits growth factor-like activity by recruiting resident stem cells to facilitate tissue repair through CXCR4 activation [41], while its proinflammatory activity is actively suppressed through interactions with CD24 [36] and TIM-3 [37]. Notably, HMGB1 drives both the migration and proliferation of vessel-associated stem cells to promote skeletal muscle regeneration via a RAGE-dependent mechanism [42] and accelerates re-epithelialization during intraoral palatal wound healing [43]. Whether these anti-inflammatory and pro-regenerative activities are governed strictly by extracellular HMGB1 concentration, or additional microenvironmental factors, such as local redox status and receptor availability, are involved, remains to be elucidated.

### 2.5. Age-Dependent Upregulation of HMGB1 Release

HMGB1 is widely recognized as a molecular signature of aging, with physiological aging driving increased HMGB1 expression and extracellular secretion in the brain. Specifically, senescent fibroblasts secrete oxidized HMGB1, i.e., dsHMGB1, which induces cytokine production via TLR4 signaling [44]. HMGB1 expression in the hippocampus of aged (24-month-old) rats, as well as its serum levels, is significantly elevated [45]. Similarly, total HMGB1 levels are significantly increased in the frontal cortex, striatum, and serum of aged (7.5-month-old) wild-type mice compared to young (3.5-month-old) controls [5], indicating an age-dependent upregulation of HMGB1 across both central and peripheral compartments.

Cellular senescence is characterized by the upregulation of conventional pro-inflammatory senescence-associated secretory phenotype (SASP) factors (such as IL-1α, IL-6, IL-8, and MMP-3) along with HMGB1, which are closely linked to aging. These factors, independently or synergistically, impair homeostatic microglial function and genomic integrity, driving chronic neuroinflammation and neural dysfunction [2,46]. Notably, extracellular HMGB1 has been proposed to mediate both stress- and age-induced neuroinflammatory priming in microglia [47]. Specifically, rats exposed to an acute physiological stressor, such as an inescapable tail-shock, maintained baseline brain cytokine levels under resting conditions. In contrast, a subsequent LPS challenge 24 h later induced a disproportionately severe neuroinflammatory response, characterized by elevations in IL-1β, IL-6, and TNFα, compared to non-stressed, LPS-treated controls [48]. However, blocking extracellular HMGB1 signaling with the HMGB1 A-box peptide abolished this stress-induced sensitization of microglial inflammatory responses [48]. Consistently, intra-cisterna magna (ICM) administration of disulfide HMGB1 (dsHMGB1) alone was sufficient to sensitize microglia to exaggerated proinflammatory activation following subsequent LPS treatment [48,49]. The concept of microglial ‘priming’ is fundamentally rooted in innate immunity, referring to a state where an initial stimulus, such as an infection or injury, induces persistent, sub-threshold molecular alterations that sensitize the cells to future insults. However, because stress and aging profoundly reshape the genomic and epigenetic landscape [32], whether extracellular HMGB1 acts as a distinct, specialized priming mediator or merely serves as an important, non-specific adaptive factor in the brain remains debatable and requires further clarification.

### 2.6. Transcriptional Upregulation and Enhanced Secretion of HMGB1 in the AD Brain

Clinical studies demonstrate that HMGB1 levels are significantly elevated in both the cerebrospinal fluid (CSF) [4] and serum [50,51] of patients with mild cognitive impairment (MCI) or AD. Furthermore, patient-specific data reveal a positive correlation between Aβ load and HMGB1 levels [51]. Consistently, post-mortem analyses confirm that HMGB1 protein is robustly upregulated in AD brain tissue relative to age-matched controls [8,52]. Animal studies revealed distinct regional variations in HMGB1 expression and distribution throughout the brain. Specifically, the frontal cortices of Tg-APP/PS1 mice exhibit a significant reduction in HMGB1 levels with a concurrent surge in circulating serum, whereas its expression levels in the striatum remain stable [5]. These data suggest that HMGB1 depletion occurs differentially across brain regions, likely reflecting a more pronounced pathological translocation of HMGB1 in the frontal cortex compared to the striatum. Gaikwad et al. demonstrate that pathological tau oligomers triggers astrocyte senescence through HMGB1 release, which correlates with markedly reduced HMGB1 levels in the frontal cortex of hTau-expressing transgenic mice [53]. Furthermore, tau oligomer-associated astrocytes display a senescence-like phenotype in the brains of patients with AD and frontotemporal dementia (FTD) [53]. These findings underscore the importance of investigating the physiological significance of the interplay between HMGB1 and tau pathology in the progression of AD.

It is intriguing to note that serum HMGB1 levels in individuals with MCI exceed those found not only in cognitively unimpaired controls, but also in patients with established AD [51]. Aligning with these findings, HMGB1 levels in the CSF are significantly higher in the MCI group than in the AD group. Specifically, in the MCI group, there is a positive correlation between levels of CSF-HMGB1 and Aβ42 and Aβ40 [54]. This dynamic surge may reflect active pathologic changes during early-stage AD pathogenesis. For example, the proportion of active necrosis increases during the preclinical stage in 5xFAD mice (1 to 6 months of age) and subsequently declines between 6 and 12 months of age as cognitive deficits manifest. The temporal dynamics of necrotic cell death may account for the transient elevation of CSF-HMGB1 reported in patients with MCI [54]. Further longitudinal studies are required to fully validate HMGB1 as a potential biomarker.

## 3. HMGB1-Mediated Pathophysiology in AD

Accumulating evidence indicates that extracellular HMGB1 drives Aβ pathogenesis through multiple integrated pathways, including the exacerbation of neuroinflammation, impairment of microglial Aβ clearance, acceleration of synaptic degeneration, and compromise of genomic stability.

### 3.1. HMGB1-Driven Vicious Cycle of Neuroinflammation and Aβ Pathology

**Figure 2:**
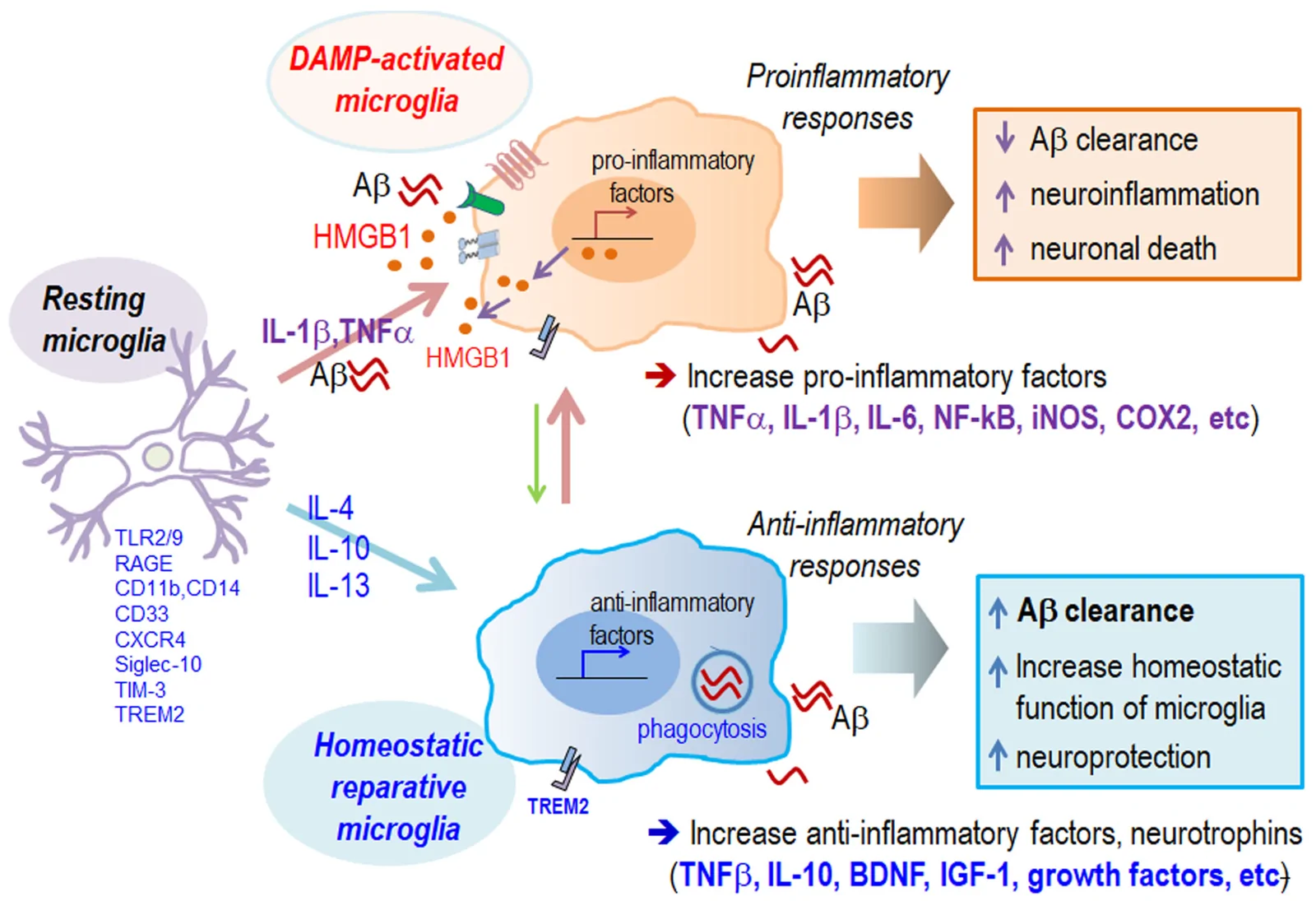
HMGB1 acts as a DAMP, inducing diverse pathologic effects in the AD brain. Resting microglia remain vigilant and can be activated by physiological levels of Aβ or anti-inflammatory cytokines (e.g., IL-4, IL-10, IL-13). Upon activation, they transition into homeostatic, reparative microglia. In this state, microglia promote Aβ clearance through phagocytosis, enhance homeostatic and tissue-repair functions, and confer neuroprotection. Conversely, increased Aβ accumulation or elevated levels of inflammatory factors (such as TNFα, IL-1β, IL-6) induce the release of HMGB1. Extracellular HMGB1 stimulates damage-associated molecular pattern (DAMP) receptors (e.g., TLR4 and RAGE), driving heightened neuroinflammatory responses. This triggers the further release of pro-inflammatory cytokines, perpetuating a vicious cycle of neuroinflammation and sustained HMGB1 release. DAMP-activated microglia exhibit impaired Aβ clearance, induce synaptic dysfunction, and exacerbate AD pathogenesis.

Chronic neuroinflammation is a pivotal driver of AD pathogenesis, sustained from disease onset through late-stage progression. Accumulating Aβ and pathological tau induce neuroinflammatory responses by triggering pro-inflammatory cascades in resident CNS immune cells. Activated microglia secrete pro-inflammatory cytokines (TNF-α, IL-1β, IL-6) and HMGB1 [2]. Extracellular HMGB1 stimulates a diverse array of microglial surface receptors, such as TLR2/4, RAGE, and Mac-1 (CD11b/CD18), thereby propagating multiple downstream signaling cascades (Figure 2). The resulting chronic neuroinflammatory conditions compromise the structural and functional integrity of neighboring neurons. This subsequent neuroaxonal damage perpetuates a pathogenic feedback loop that accelerates AD progression, positioning HMGB1 as both a primary initiator and a potent amplifier of the neuroinflammatory cascade.

Aβ42 oligomers induce the nucleocytoplasmic translocation of HMGB1 and its subsequent extracellular secretion in the BV2 microglia cell line [55] and in primary microglia cultures [5]. Aβ (25–35) treatment in BV2 cells impairs the mitochondrial SIRT3/SOD2 pathway, leading to heightened oxidative stress, hyperacetylation of HMGB1, and its subsequent extracellular release [56]. Aβ oligomers induce HMGB1 secretion in primary cortical neuronal cultures [4,5], in primary hippocampal neuronal cultures [15], and in primary microglia cultures [5]. Aβ-induced HMGB1 release from microglia appears to be much higher than that from neurons [5]. Collectively, these studies indicate that Aβ triggers both neurons and microglia to secrete HMGB1, which in turn drives a vicious cycle of neuroinflammation and amyloid pathology.

### 3.2. HMGB1 Acts as a DAMP, Engaging Distinct Receptor Signaling

Both HMGB1 and Aβ act as DAMPs that induce inflammatory responses through shared and distinct membrane receptors (Figure 2). Several recent reviews provide an extensive summary of the receptors activated by either HMGB1 or Aβ and their corresponding downstream signaling pathways [6,57]. The receptors activated by both HMGB1 and Aβ include TLR2/4, RAGE, CD14, and scavenger receptor A [6,57]. Mechanistically, activation of these receptors triggers downstream signaling pathways, most notably the MAPK and NF-κB cascades.

Among these receptors, TLR2/4 and RAGE are well-characterized to mediate HMGB1- and Aβ-induced inflammatory responses. Extracellular activity of HMGB1, particularly the disulfide isoform (dsHMGB1), binds to the TLR4/MD-2 complex to induce the secretion of pro-inflammatory cytokines [27]. In 5xFAD transgenic mice, the HMGB1/TLR4 axis drives neurite degeneration by stimulating the downstream phosphorylation of MARCKS (myristoylated alanine-rich C-kinase substrate) [4]. Similarly, RAGE mediates HMGB1- and Aβ-induced neuroinflammation, thereby accelerating AD pathogenesis [6]. While acute activation of TLR4 and RAGE stimulates the canonical NF-κB pathway, sustained HMGB1 exposure sensitizes this pro-inflammatory response through NF-κB-mediated priming of the NLRP3 inflammasome in microglial cells [48,49]. Furthermore, Aβ exposure significantly upregulates the secretion of the complement component C1q and macrophage colony-stimulating factor (M-CSF) in cultured human microglia, and this response was heightened in microglia derived from AD patients compared to non-demented controls [58]. The complement system initially enhances acute inflammation to recruit phagocytes for the clearance of Aβ and cellular debris in the brain. However, chronic activation of the complement system and prolonged immune infiltration exert deleterious effects [57], driving aberrant, microglia-mediated synaptic pruning and subsequent neuronal dysfunction in AD mouse models [59,60]. This microglial activation is further exacerbated by extracellular HMGB1, which triggers a feedforward loop of neuroinflammation and synapse elimination in mice by stimulating the microglial secretion of classical complement proteins [61].

### 3.3. HMGB1-Mediated Stabilization of Toxic Aβ Oligomers

HMGB1 directly interacts with Aβ, thereby stabilizing its neurotoxic oligomeric forms. Histological analyses indicated the co-localization of HMGB1 with Aβ, leading to a significant accumulation of HMGB1 within senile plaques in AD-like brains [8,62]. Consistent with these findings, Fujita et al. demonstrated that HMGB1 promotes Aβ oligomerization, whereas neutralizing HMGB1 with a monoclonal antibody suppresses its oligomerization [4]. Aβ oligomers exhibit far higher cytotoxicity than their monomeric or fibrillar counterparts [63,64]. By stabilizing these toxic oligomeric species, HMGB1 appears to potentiate Aβ-mediated synaptotoxicity, acting as a parallel pathogenic driver, alongside HMGB1-dependent neuroinflammation. Thus, HMGB1 significantly dysregulates cellular function through both Aβ-independent and Aβ-mediated synergistic pathways, both of which exert profound neurotoxic effects.

### 3.4. HMGB1 Impairs Microglial Aβ Clearance

Under normal physiological conditions in the brain, Aβ is generated as a byproduct of normal neuronal metabolism and APP cleavage, but its steady-state concentration remains low due to efficient clearance mechanisms, including enzymatic degradation extracellularly, microglial phagocytosis, and trans-vascular efflux [65,66,67]. Consequently, impaired clearance relative to production causes the accumulation of Aβ, driving the neuropathology observed in AD brains. Using metabolic labeling, Mawuenyega et al. quantified the production and clearance rates of Aβ42 and Aβ40 in the human CNS, revealing that while production rates were comparable between AD patients and cognitively normal controls, clearance rates were significantly impaired in the AD group [68]. These data underscore the pivotal role of Aβ clearance in AD pathogenesis.

Extracellular HMGB1 impairs the brain’s primary phagocytic activity, leading to accelerated accumulation of Aβ. In macrophages, HMGB1 impairs efferocytosis via binding to the αvβ3-integrin [69] and suppresses the phagocytosis of apoptotic neutrophils by interacting with phosphatidylserine [70]. Consistently, exogenously applied HMGB1 suppresses the phagocytosis of Aβ in primary rat microglial cultures [8]. Furthermore, the injection of HMGB1 into the rat hippocampus delayed Aβ42 clearance, and exacerbated neuronal loss in Aβ42-injected rats [71]. HMGB1 co-localizes with Aβ plaques, which are surrounded by activated microglia in the brains of both AD patients [8,62] and animal models [4,5] (Figure 1b). The pivotal role of HMGB1 in modulating Aβ phagocytosis was further corroborated utilizing HMGB1 inhibitors such as neutralizing antibodies [4]. The loss-of-function approach, together with the complementary gain-of-function approach, extends our understanding of how extracellular HMGB1 directly suppresses general microglial phagocytosis. However, because HMGB1 can form a complex with Aβ, it remains to be elucidated whether this interaction directly impairs Aβ internalization or delays its clearance within microglial phagolysosomes.

Microglial Aβ clearance involves multiple distinct receptor networks, encompassing pattern recognition receptors for danger signals (TLR2, TLR4, RAGE) [72,73] and scavenger receptors [74]. Beyond conventional phagocytosis, microglia can engage alternative strategies such as digestive exophagy to process bulky Aβ aggregates [75]. However, microglial Aβ clearance is severely compromised by Aβ itself and its downstream induction of extracellular HMGB1. Chronic inflammation induced by these factors downregulates key phagocytic and scavenger receptor complexes, thereby diminishing microglial phagocytic activity and leading to Aβ accumulation in AD brains [4,6].

TREM2 (Triggering Receptor Expressed on Myeloid Cells 2) is a critical regulator of microglial phagocytosis. TREM2 binds to Aβ oligomers, whereas AD-associated mutations in TREM2 (e.g., TREM2-R47H or R62H) significantly diminish its Aβ-binding ability, impairing microglial activation and reducing Aβ clearance [76,77]. Notably, these mutations increase AD risk by 1.5- to 4-fold [78]. In line with this finding, transgenic mouse models of AD (such as 5xFAD and APP/PS1) lacking TREM2 or carrying the TREM2-T66M mutation exhibit decreased microglial clustering around plaques, impaired Aβ clearance, and enhanced amyloid deposition [77,79]. Collectively, these findings highlight TREM2 as a key receptor required for effective phagocytic clearance of Aβ by microglia. Given the critical role of TREM2 in microglial homeostatic function, the Aβ- and HMGB1-dependent regulation of TREM2 expression and its specific contributions to microglial phagocytosis remain understudied and warrant further investigation.

Beyond phagocytic uptake, both microglia and neurons employ an alternative Aβ clearance pathway mediated by the enzymatic degradation. The major enzymes responsible for enzymatic clearance include neprilysin (NEP), insulin-degrading enzyme (IDE), matrix metalloproteinases (MMPs), and tissue-type plasminogen activator (tPA) [65]. These Aβ-degrading enzymes are widely expressed throughout the brain, with distinct cell-type-specific expression profiles. Neurons express and utilize NEP, IDE, MMP2/9, and tPA, while microglia primarily rely on IDE and MMP9 to clear extracellular Aβ [66,80,81]. The expression of these enzymes is significantly reduced in AD brains [80,81]. Although the Aβ- and neuroinflammation-dependent precise regulatory mechanisms governing these extracellular proteinases in AD remain unresolved, whether this downregulation is a direct consequence of neuroinflammation activated by Aβ and HMGB1 needs further study.

Parallel to cellular and enzyme-mediated pathways, the brain utilizes multiple trans-vascular pathways to remove Aβ and metabolic waste. A glia-dependent perivascular network, called the glymphatic system, serves as the major route for these trans-vascular processes [67]. Neuroinflammation impairs glymphatic function and reduces CSF flow [82]. Aquaporin-4 (AQP4) is a key mediator of this clearance pathway; its altered expression or mis-localization in perivascular astrocytes directly correlates with neuroinflammation and AD pathology [83]. Although the glymphatic system plays a major role in Aβ clearance and shows reduced activity in AD-like brains, the mechanisms linking its impairment to Aβ- and HMGB1-related neuroinflammation remain poorly understood. Specifically, whether Aβ or HMGB1 signaling directly targets the astroglial network or indirectly disrupts glymphatic function through microgliosis remains to be determined.

### 3.5. Extracellular HMGB1 Induces Synaptic Degeneration and Cognitive Impairment

Normally, the actin cytoskeleton stabilizes postsynaptic dendritic spines by anchoring membrane proteins and maintaining plasma membrane integrity. MARCKS is a membrane-associated protein involved in the structural modulation of the actin cytoskeleton, cell motility, and phagocytosis. HMGB1/TLR4-mediated phosphorylation of MARCKS at Ser46 reduces its affinity for both actin and membrane phosphatidylinositol 4,5-bisphosphate (PIP2), leading to spine destabilization and subsequent cognitive impairment in 5xFAD transgenic mice [4]. Notably, this specific MARCKS phosphorylation precedes histologically detectable Aβ aggregation [84], strongly implying that synaptic and neurite degeneration occurs early in AD pathogenesis.

While synaptic pruning is a normal developmental process, microglia play a role in the refinement through targeted phagocytic engulfment [85]. The classical complement cascade (C1q/C3) mediates aberrant synapse elimination by both microglia and reactive astrocytes in J20 Tg-APP/PS1 mice [59]. A similar synapse engulfment by microglia and reactive astrocytes was also detected in tauopathy-mimicking Tau-P301S mice [60]. Notably, HMGB1 induces excessive pruning of synapses in animal models of sepsis-associated encephalopathy [86] and of depression [61]. Mechanistically, dsHMGB1 activates microglia and upregulates C1q/C3-dependent synaptic elimination [61]. Consistent with these reports, HMGB1 A-box administration reduces synaptic degeneration and rescues behavioral deficits in AlCl_3_/D-gal-induced AD models [87].

Extracellular HMGB1 exacerbates cognitive impairment in transgenic AD models. Mechanistically, intracerebroventricular injection of recombinant HMGB1 aggravates memory deficits, whereas this effect can be neutralized by inhibiting RAGE and TLR4 signaling in a mouse model of AD [6,88,89]. Furthermore, extracellular HMGB1 drives pathological synaptic loss and cognitive decline in animal models of traumatic brain injury [90] and sepsis-associated encephalopathy [86,91]. Collectively, these reports suggest that HMGB1-induced neuroinflammation and synaptic degeneration may represent a convergent neuropathological mechanism driving cognitive dysfunction in AD.

### 3.6. HMGB1 Regulates DNA Repair and Genomic Stability in AD

HMGB1 is a DNA-binding chaperone that maintains chromatin integrity and actively participates in DNA repair [92]. Emerging evidence indicates that nuclear HMGB1 depletion impairs DNA repair mechanisms, ultimately undermining genomic integrity in AD brains. DNA double-strand breaks (DSBs) occur naturally during physiological neuronal activity, but their levels are significantly elevated by neuronal hyperactivity and are further exacerbated by Aβ [93]. Diverse forms of DNA lesions are detected in AD patients and animal models. Post-mortem analyses of the hippocampus and frontal cortex have revealed a significant accumulation of DNA DSBs in both neurons and astrocytes of patients with AD or MCI, compared with age-matched controls [94]. Extracellular HMGB1 induces TLR4-mediated phosphorylation of Ku70 (a critical DSB repair protein), disrupting its affinity for DNA DSB sites. The resulting impairment in Ku70-mediated DSB repair undermines genomic integrity, culminating in atypical cell death [95]. However, administering an anti-HMGB1 monoclonal antibody to AD model mice reduces neuronal DSBs, suppresses atypical cell death and ameliorates cognitive symptoms [95]. Collectively, these findings establish HMGB1 as a critical regulator of genomic stability and DNA repair pathways in AD brains.

## 4. Therapeutic Potential of HMGB1 Inhibitors in AD Animal Models

Various classes of HMGB1 inhibitors have been developed and categorized by their distinct mechanisms of action, ranging from small molecules preventing its release to agents that block its interaction with inflammatory receptors. Glycyrrhizin (glycyrrhizic acid) is a small molecule that directly binds to HMGB1 [96,97]. Ethyl pyruvate inhibits the acetylation and release of HMGB1 via effects on SIRT1/STAT signaling [98]. Anti-HMGB1 monoclonal antibodies and HMGB1 A-box peptide were developed and used to specifically target HMGB1 [4,87,99,100].

HMGB1-targeting interventions have demonstrated notable improvements in specific pathological features and behavioral deficits across animal models of AD. Specifically, administration of an anti-HMGB1 antibody to 5xFAD Tg-APP/PS1 transgenic mice for three months (from 3 to 6 months of age) inhibited neurite degeneration, reduced neuronal death, enhanced microglial Aβ phagocytosis, and rescued cognitive dysfunction [4]. HMGB1-targeting biologics, such as HMGB1 Box A peptides, short peptide inhibitors, and siRNA/shRNA constructs, have not yet been reported in the context of AD. However, their neuroprotective and anti-inflammatory effects have been established in cell cultures and post-ischemic stroke models of neuroinflammation-driven cell death [15,100,101,102], demonstrating their translational promise. In addition, gene therapy delivering an HMGB1 A-Box-expressing plasmid suppresses microgliosis, reduces hippocampal Aβ accumulation, and reverses memory loss in AlCl_3_ & D-gal-induced AD-like rat models [87]. These studies demonstrate the therapeutic potential of HMGB1-targeted biologics.

Administration of ethyl pyruvate and glycyrrhizin for 8 weeks in transgenic mice overexpressing hTau reduced neurofibrillary tangle deposition and Tau phosphorylation, thereby improving neuronal cell loss and cognitive performance in Y-maze and novel object recognition tests [53]. Administration of diammonium glycyrrhizin in Aβ-42-injected mice reduced oxidative stress and mitochondrial dysfunction and improved cognitive deficits [103]. Intragastric administration of trilobatin (4, 8 mg· kg^−1^ d^−1^) for 3 months in APPswe/PS1dE9 transgenic mice suppressed the HMGB1/TLR4/NF-κB pathway by targeting HMGB1 and activating SIRT3/SOD2 signaling and rescued cognitive impairment [56]. Altogether, although small-molecule inhibitors and biologics targeting HMGB1 pathways demonstrate significant therapeutic potential, translating these strategies into clinical AD interventions requires rigorous validation across specific disease stages and pathophysiological contexts.

## 5. Conclusions and Perspectives

AD pathogenesis involves a complex biological interplay with HMGB1, which exerts compartmentalized functions ranging from nuclear genomic stabilization to extracellular neuroinflammatory toxicity. Extracellular HMGB1 induces inflammatory responses through various unrelated surface receptors. Furthermore, HMGB1-associated dysregulation occurs during normal brain aging, and is exacerbated by Aβ and other pathological stressors. Therefore, a comprehensive understanding of HMGB1’s distinct mechanisms of action at various levels is critical for effectively controlling AD pathology.

Regarding the molecular and functional domains of HMGB1, the A-box and B-box are often conceptualized as anti-inflammatory and pro-inflammatory, respectively. While this holds true in certain contexts, it is critical to note that within the intact, full-length HMGB1 protein, the A-box domain does not override or neutralize the pro-inflammatory activity of the B-box domain. Native extracellular full-length HMGB1 functions as a predominantly pro-inflammatory mediator. Furthermore, no experimental evidence suggests that the A-box and B-box domains exert autonomous, opposing immunomodulatory actions within the tertiary structure of the intact protein, making such a functional distinction irrelevant in vivo. Nevertheless, this functional dichotomy becomes evident when these domains act as independent peptide fragments, providing a versatile molecular platform for the design of targeted interventions.

Although small-molecule and phytochemical HMGB1 inhibitors are currently available, utilizing HMGB1-derived peptides to disrupt downstream signaling provides a highly targeted alternative for therapeutic modulation. From this perspective, other members of the HMGB family also merit attention. While HMGB1 is ubiquitously expressed across almost all cells and tissues, HMGB2 expression is predominantly restricted to lymphoid organs and the thymus. Meanwhile, HMGB3 is mainly expressed in hematopoietic stem cells (HSCs) and early hematopoietic progenitor cells, and HMGB4 is primarily distributed in the testes and neurons [104]. All isoforms in the HMGB family exhibit highly conserved sequences and share similar functional roles across mammals. The extent of their functional redundancy within identical cellular or tissue microenvironments needs to be characterized further. While the primary structures of HMGB isoforms offer valuable templates for engineering peptide-based modulators of HMGB1, critical questions persist. Specifically, it remains to be evaluated whether isoform-specific biologics are necessary for treating AD and whether systemic administration avoids off-target systemic toxicity.

Serum HMGB1 levels are elevated in aged animals (7.5 months of age) compared to young controls (3.5 months of age) and are further enhanced in age-matched Tg-APP/PS1 mice [5]. Similarly, Tanaka et al. reported that HMGB1 levels are significantly increased in the CSF of patients with MCI, though this elevation is less pronounced in those with AD [54]. Consistent with these findings, active neuronal necrosis, indicated by pSer46MARCKS, peaks during the preclinical stages of AD mouse models and human MCI patients [54]. Collectively, these data raise the possibility that serum or CSF HMGB1 could serve as a biomarker for staging AD pathogenesis. While HMGB1 represents a promising mechanistic target for controlling AD-related neuroinflammation and Aβ pathology, the clinical efficacy, safety, and readiness of HMGB1-targeted approaches for use in patients with AD have not yet been established, and further studies are required to establish its clinical utility as a biomarker.

## Data Availability

No new data were created or analyzed in this study. Data sharing is not applicable to this article.

## Abbreviations

The following abbreviations are used in this manuscript.

AD	Alzheimer’s disease
HMGB1	High mobility group box 1
DAMP	Damage-associated molecular pattern
APP	Amyloid precursor protein
PS1	Presenilin 1
Aβ	Amyloid-β
TLR	Toll-like receptor
RAGE	Receptor for Advanced Glycation End products
NLSs	Nuclear localization signals
CXCR4	C-X-C motif chemokine receptor 4
MARCKS	Myristoylated alanine-rich C-kinase substrate
MCI	Mild cognitive impairment
FTD	Frontotemporal Dementia
TREM2	Triggering receptor expression on myeloid cells 2
CSF	Cerebrospinal fluid
PrxI/II	Peroxiredoxins I and II
SIRT1	Sirtuin1
HAT	Histone acetyltransferase
HDAC	Histone deacetylase
NOD2	Nucleotide-binding oligomerization domain-containing protein 2
CD11b	Cluster of Differentiation 11b
TIM-3	T-cell Immunoglobulin and Mucin domain-containing protein 3
SASP	Senescence-associated secretory phenotype
MMP-3	Matrix Metalloproteinase-3
ICM	Intra-cisterna magna
SOD2	Superoxide Dismutase 2
M-CSF	Macrophage colony-stimulating factor
NEP	Neprilysin
IDE	Insulin-degrading enzyme
tPA	Tissue-type plasminogen activator
AQP4	Aquaporin-4
PIP2	Phosphatidylinositol 4,5-bisphosphate
